# Efficiency of a Sensory-Adapted Dental Environment Versus Regular Dental Environment in Neurotypically Healthy Children: A Parallel-Arm Interventional Study

**DOI:** 10.7759/cureus.62109

**Published:** 2024-06-10

**Authors:** Ayesha Fathima, Mahesh R, Ramesh R, Kiran K Pandurangan

**Affiliations:** 1 Pediatric and Preventive Dentistry, Saveetha Dental College and Hospitals, Saveetha Institute of Medical and Technical Sciences, Saveetha University, Chennai, IND; 2 Prosthodontics, Saveetha Dental College and Hospitals, Saveetha Institute of Medical and Technical Sciences, Saveetha University, Chennai, IND

**Keywords:** sensory adaptive dental environment, non-pharmacological management, multisensory environment, behaviour management, anxiety scale

## Abstract

Introduction

The basic principle of a sensory adaptive dental environment is that an individual's sensory experiences have a significant impact on their emotional and psychological well-being. Taste, smell, touch, hearing, and sight are the five basic senses that affect our perception and responses to the environment. The study aimed to assess the effectiveness of a Sensory-Adaptive Dental Environment (SADE) compared with a Regular Dental Environment (RDE) in reducing anxiety, improving behavior, and providing a smooth experience for children undergoing dental treatment.

Materials and methods

This parallel-arm pilot study was conducted at the outpatient Department of Pediatric and Preventive Dentistry, Saveetha Dental College and Hospitals, Chennai, from January 2024 to March 2024. A total of 148 children who met the inclusion criteria were divided into two groups: Group I (intervention group) received SADE or MSE (Multi-Sensory Environment) intervention, while Group 2 (control group) underwent dental treatments in a Regular Dental Environment (RDE). Patient behavior was assessed using Frankl's behavior rating scale, and anxiety levels were measured using Ayesha's Oddbodd anxiety scale. Additionally, heart rate and oxygen saturation (SpO_2_) were evaluated using a pulse oximeter. Statistical analysis was conducted using IBM SPSS Statistics for Windows, Version 26.0 (IBM Corp., Armonk, NY), with significance set at a p-value less than 0.05.

Results

Before the procedure, there were no notable differences in behavior or anxiety levels. However, after the procedure, children undergoing treatment under SADE resulted in markedly improved behavior and notably lower anxiety levels. Also, this correlated with reduced anxiety levels, indicated by lower heart rates and higher oxygen saturation levels.

Conclusion

The study concluded that there were notable differences in patient experiences between SADE and RDE. After their dental procedures, participants in the SADE group were found to behave better and feel less nervous. Still, in the conventional setting, only improved behavior was noted, with no significant difference in anxiety levels. Overall, our study suggests that dental offices can significantly enhance patient experiences by providing a sensory-friendly setting that helps children feel more at ease, improves patient outcomes, and less nervous during their visits.

## Introduction

As a pediatric dentist, the conventional approach to oral healthcare is hindered by dental phobia, with pain directly influencing anxiety levels and subsequently affecting a child's conduct. Accurate assessment of the child's stage of development, dental fear, and apprehension, in anticipation of treatment termed anticipatory dental anxiety, plays an important role in child care in dentistry [1]. Effective communication strategies judicious command usage and factors like previous negative dental visits or parenting techniques influence a child's reluctance to cooperate during dental appointments [2].

Behavior guidance describes a patient-dentist relationship that is ongoing, focused on establishing communication, and ensures the child's and oral health providers' safety while delivering dental care [3]. Over the years, several behavior guidance techniques involving sensory elements have been implemented, and in modern dentistry, the sensory-integrated approach has become the need for the hour as pediatric dentists must be skilled in a range of behavior guidance techniques to meet the needs of each child, as well as be tolerant and adaptable in their application, due to the wide range of attitudes and temperaments that accompany children's diverse physical, intellectual, emotional, and social development [4,5].

Sensory-Adaptive Dental Environment (SADE) based on the core idea that a person's emotional and psychological health are greatly influenced by their sensory experiences is an innovative development that has gained popularity [6]. The five main senses, taste, smell, touch, hearing, and sight, influence how we perceive and react to the environment [7]. The American Academy of Pediatric Dentistry (AAPD) in behavior guidance for pediatric dental patients 2020, has included SADE and Animal-assisted therapy as additional considerations for dental patients with anxiety or special healthcare needs [8].

By transforming the conventional dental setting and promoting a more welcoming and comfortable environment, we identify and meet the various sensory needs of patients for a comfortable and hassle-free dental experience [9]. When one takes into account the wide range of people who might encounter increased sensitivity or sensory difficulties during dental visits, examples include people with autism spectrum disorders, people with sensory processing disorders, and people with anxiety disorders [10]. These significant populations can benefit from the changes in the regular environment for their dental treatments. 

Regular dental settings have been associated with unsettling atmospheres that are defined by clinical sterility, glaring lights, and the whirring noises of dental equipment. For people who are sensitive to certain stimuli, these environments can be more upsetting. This could make them more stressed out, reluctant to visit the dentist, or even delay necessary treatments [11]. The need for a more compassionate and individualized approach to child healthcare with a specialized environment that influences the children has given rise to the idea of SADE. Using this specialized environment, dental offices can establish a setting wherein it reduces anxiety, encourages relaxation, and ultimately improves the child's experience of the dental atmosphere by components that influence the sensory preferences of children [12].

Children on the autism spectrum suffer from difficulty in sensory processing. Anything outside their environment that is unfamiliar and too loud visually or auditorily can overwhelm them. The SADE is designed primarily for children with autism [13]. However, there is a lack of research studies involving neurotypically healthy children making it a unique initiative in this study. This study was conducted to assess the effectiveness of SADE compared with the Regular Dental Environment (RDE) in children based on reducing anxiety, improving child behavior, and providing a smooth experience for children undergoing dental treatment.

## Materials and methods

Study setting

The present parallel-arm pilot study was done from January 2024 to March 2024 at the outpatient Department of Pediatric and Preventive Dentistry, Saveetha Dental College and Hospitals, Chennai, India.

Study population and size

**Figure 1:**
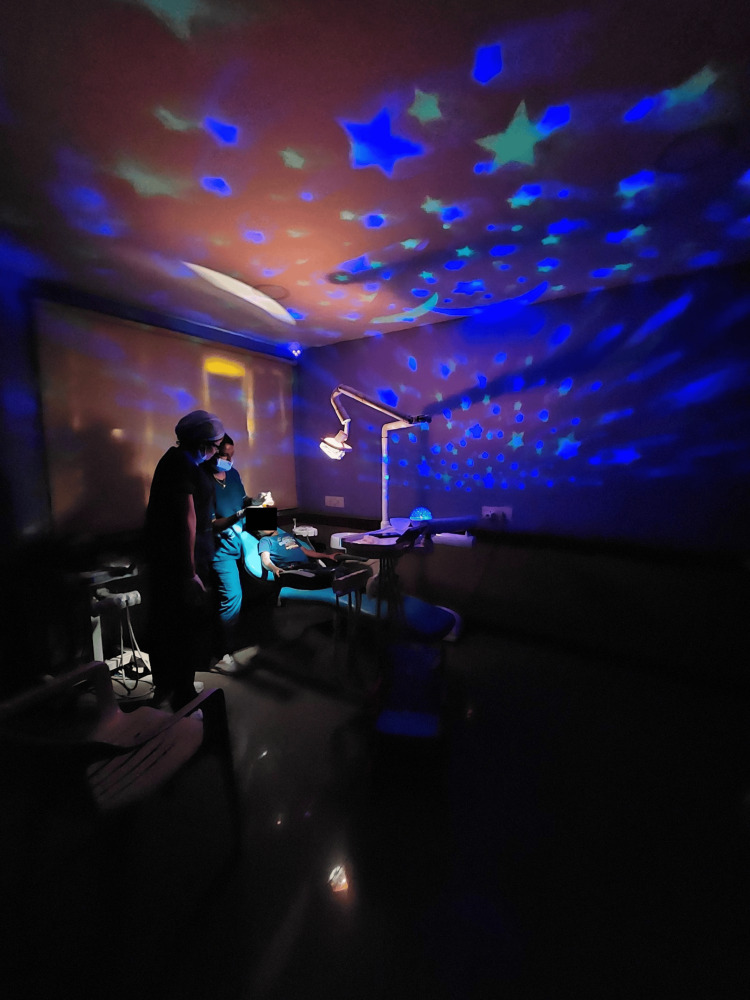
Sensory-adaptive dental environment

**Figure 2:**
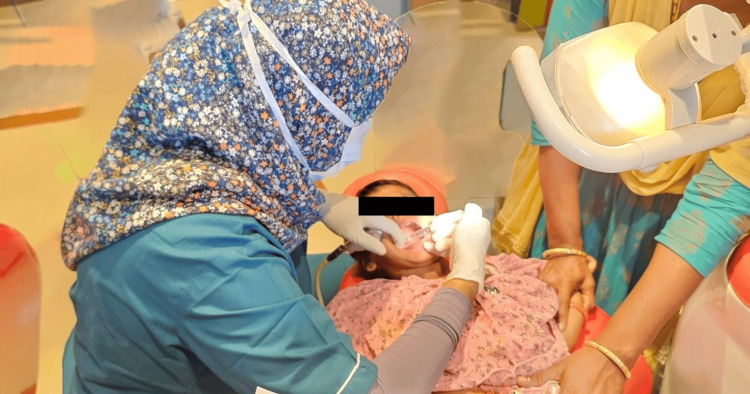
Regular dental environment

The study consisted of 148 neurotypically healthy children who understand and speak the native language Tamil. The study size was divided into two groups with an allocation ratio of 1:1 (n=74). The participants were divided into the intervention group (SADE) (Figure 1) and the control group (RDE) (Figure 2).

Ethical clearance 

Before participating in the trial, patients are provided with comprehensive information about the study, including its purpose and procedures. Access to patient data is restricted to only those individuals directly involved in the research and the participant's anonymity was maintained. The study commenced after obtaining approval from the Institutional Human Ethical Committee (IHEC), Saveetha Dental College and Hospitals, Chennai with approval number IHEC/SDC/PEDO-2104/23/081. The participant's parents or guardians provided written informed permission.

Inclusion criteria

Children between the age group of four to seven years who require extraction of mandibular primary first molar due to grossly decayed or non-restorable dental caries. Children who fall under categories II and III on Frankl's behavior rating scale.

Exclusion criteria

Children with systemic and intellectual conditions. Parents who did not give consent to the study. Children who were phobic to dark and had claustrophobia. Children who underwent procedures under pharmacological behavioral management.

Randomization

Following selection criteria, children were assigned into two groups based on the computerized block randomization approach based on the kind of dental environment setting in which they would receive their dental care

Survey instrument

The present study assessed four parameters. The patient’s behavior was assessed by Frankl’s behavior rating scale. Frankl 1 is definitely negative and includes crying violently, being afraid, or exhibiting any other signs of strong negativism. Frankl 2 is negative behaviour showing unwillingness to accept therapy, being uncooperative, and displaying mildly negative attitudes such as being withdrawn or gloomy. Frankl 3 is positive behavior where the youngster is cooperatively following the dentist's instructions while also exhibiting acceptance of treatment and being cautious when following his or her instructions. Frankl 4 is referred to as definitely positive exhibiting a strong rapport with the dentist, and an interest in dental operations [14].

**Figure 3:**
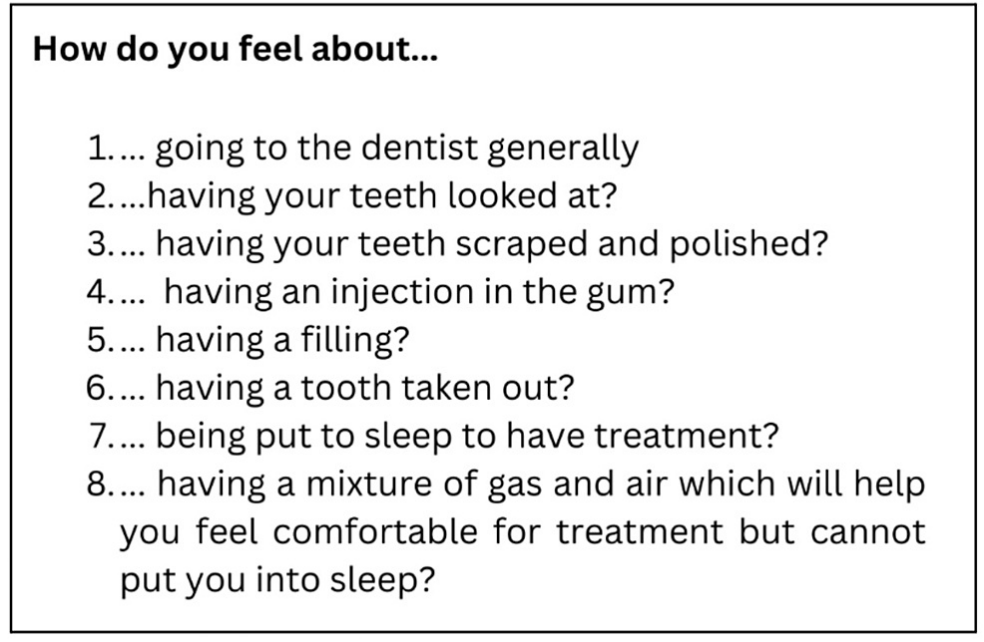
Questions of Oddbodds anxiety scale

**Figure 4:**
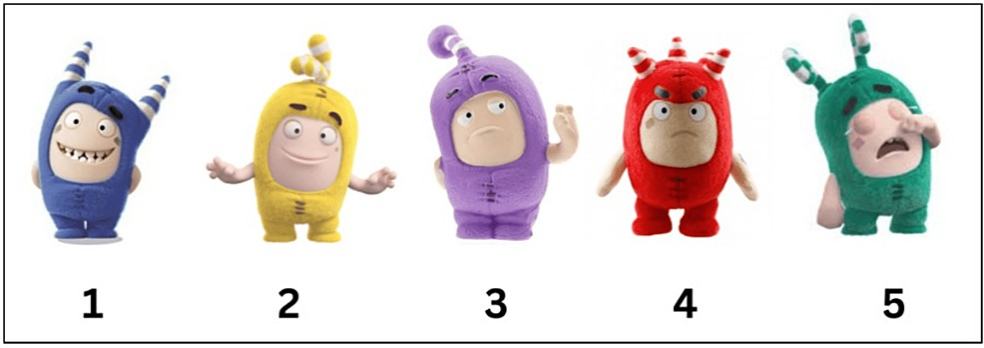
Oddbodds anxiety scale Oddbodds anxiety scale (1-5) ranging from no anxiety to severe anxiety

**Figure 5:**
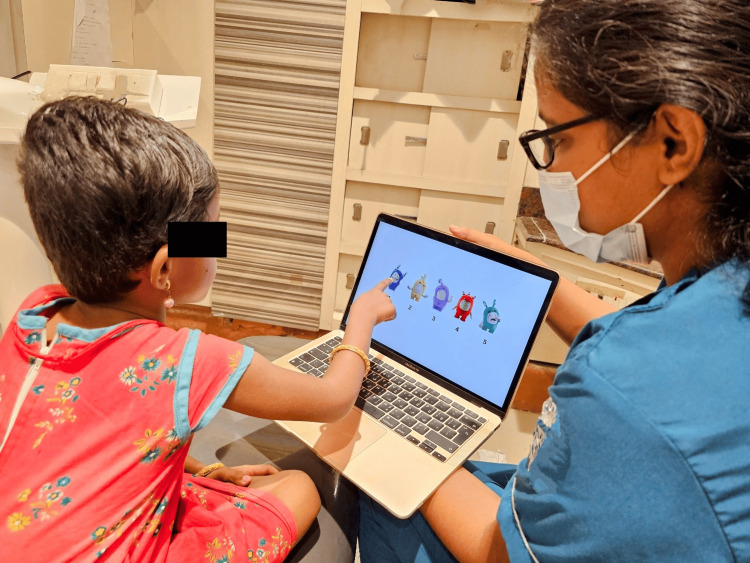
Participant choosing their anxiety level using the Oddbodds anxiety scale

The second parameter was to evaluate the anxiety by Ayesha’s Oddbodd anxiety scale [14]. Ayesha’s Oddbodd scale was designed to be a 5-point Likert scale consisting of eight prevalidated questions with the options constituting five Oddbodd cartoon characters with different emotions represented in various colors. This scale was similar to the Likert scale of 1- 5 which is interpreted as follows: score 1 corresponds to not fearful and anxious, whereas score 5 interprets severe fear and anxiety (Figures 3-5). The third and fourth parameters were to assess oxygen level through oxygen saturation (SpO_2_) level and heart rate through a pulse oximeter (Dr Trust, India) which again corresponds to the anxiety level of the child [15].

Intervention

The experimental setting for the test group in the study was designed through a series of interventions aimed at creating a soothing environment. Visual enhancements were achieved by applying glow stickers depicting a serene night sky with the moon and stars, sourced (Dream Kraft, India). Olfactory stimuli were provided through the use of a lavender oil diffuser, utilizing lavender oil from (Soulflower, India) and a diffuser (Rene-Maurice, China). To enhance the tactile experience, participants received a warm body wrap coupled with a lead jacket. Lastly, auditory elements were incorporated through the playing of calming melodies, contributing to the overall ambiance of relaxation and comfort within the SADE environment.

Data collection

The children allocated in both groups were assessed for behavior, anxiety levels, heart rate, and oxygen levels two minutes after the patient was seated for clinical examination. Then the test group children were taken to a room with a preset SADE environment and were explained about the dental procedure. Inferior alveolar nerve block, Lingual nerve block, and long buccal infiltration were administered and primary lower molar extraction was carried out. Again two minutes after the procedure, all the parameters were assessed. 

In the control group, all the previously mentioned parameters were assessed and the children were taken to the regular outpatient department where they underwent their dental procedure. Similarly, two minutes after the extraction procedure, the parameters were assessed.

Statistical analysis

The data was tabulated and analyzed in IBM SPSS Statistics for Windows, Version 26.0 (IBM Corp., Armonk, NY). The behavior and anxiety were described with frequency and percentages. Heart rate and oxygen levels were described as mean and standard deviation. Shapiro-Wilk test was used to assess the normality of the distribution. Wilcoxon sign rank test was used to assess the pre and post-differences within the group for behavior and anxiety levels. Mann-Whitney U test was used to assess the differences between the groups for the same variables. For heart rate and oxygen levels, a paired t-test was used to test the pre and post-op differences in the same group, and intergroup differences were assessed by an independent t-test with a statistical significance of less than 0.05.

## Results

The present research was done on 148 participants who were divided into two groups (n=74) according to the type of environment they underwent dental treatment. Behavior, anxiety, heart rate, and oxygen saturation levels were assessed. Behavior was assessed by Frankl’s behavior scale. Before the procedure in SADE and RDE, 43.2% had negative behavior and 56.8% had positive behavior respectively. But this changed to 39.2% positive and 60.8% definitely positive behavior after the procedure in the SADE group. In the control group, 16.2% had negative, 44.6% had positive and 39.2% had definitely positive behavior which shows behavioral changes were better in SADE (Table 1).

**Table 1:** Distribution of behavior among both the groups SADE: Sensory Adaptive Dental Environment; RDE: Regular Dental Environment

	Frankl’s behavior scale			
	Median	2 (Negative)	3 (Positive)	4 (Definitely positive)
SADE before	3	32 (43.2)	42 (56.8)	0
SADE after	4	0	29 (39.2)	45 (60.8)
RDE before	3	32 (43.2)	42 (56.8)	0
RDE after	3	12 (16.2)	33 (44.6)	29 (39.2)

Anxiety level was assessed by Ayesha’s Oddbodd scale. The median of anxiety levels before the procedure for both groups was 3. After the procedure, it reduced to 2 in the SADE group and it remained at 3 in the control group which depicts that anxiety levels were reduced in the SADE (Table 2).

**Table 2:** Distribution of anxiety levels among both the groups SADE: Sensory Adaptive Dental Environment; RDE: Regular Dental Environment

	Oddbodd anxiety scale					
	Median	1 n (%)	2 n (%)	3 n (%)	4 n (%)	5 n (%)
SADE before	3	4 (5.4)	16 (21.6)	19 (25.7)	26 (35.1)	9 (12.2)
SADE after	2	16 (21.6)	22 (29.7)	23 (31.1)	13 (17.6)	0
RDE before	3	19 (25.7)	13 (17.6)	20 (27)	10 (13.5)	12 (16.2)
RDE after	3	19 (25.7)	13 (17.6)	18 (24.3)	10 (13.5)	14 (18.9)

The mean heart rate in the SADE group was 92.31土11.44 and it reduced to 90.32土8.43. In the control group, it was 94.55土11.06 before the procedure and 97.47土5.8 after the procedure indicating that the heart rate was reduced postoperatively in the SADE whereas the heart rate increased postoperatively in RDE. This infers that the participant is relaxed and more comfortable when undergoing the procedure in SADE. Similarly, in the SADE group, SpO_2_ levels were 99.29土1.14 before the procedure and it increased to 99.45 土2.34 after the procedure. In the control group, it was 99.47土1.02 before and 98.51土1.34 after the procedure This explains there was a mild drop in the oxygen saturation in RDE (Table 3).

**Table 3:** Distribution of heart rate and oxygen saturation among both groups SADE: Sensory Adaptive Dental Environment; RDE: Regular Dental Environment; SpO_2_: Oxygen saturation

Groups	Mean 土 SD
SADE heart rate before	92.31土11.44
SADE heart rate after	90.32土8.43
RDE heart rate before	94.55土11.06
RDE heart rate after	97.47土5.8
SADE SpO_2_ before	99.29土1.14
SADE SpO_2 _after	99.45土2.34
RDE SpO_2_ before	99.47土1.02
RDEl SpO_2_ after	98.51土1.34

The Wilcoxon sign rank test, which assessed the mean differences within the group, revealed that in the SADE group, behavior significantly improved and anxiety levels significantly dropped after the procedure in the SADE environment. In the control group, behavior significantly improved after the treatment but the anxiety levels did not significantly differ (Table 4).

**Table 4:** Wilcoxon sign rank test showing significant differences in the behavior and anxiety levels before and after the treatment SADE: Sensory Adaptive Dental Environment; RDE: Regular Dental Environment; p<0.05: Statistically significant

Groups	Chi value	P value
SADE behavior before-after	-6.835	<0.001
			SADE anxiety before-after	-4.085	<0.001
RDE behavior before-after	-5.106	<0.001			
			RDE anxiety before-after	-0.345	0.730

Similarly, the parametric heart rate and oxygen levels were assessed by paired t-test. In the SADE group, heart rate was significantly reduced after the procedure but oxygen levels did not change significantly. Also in the control group, both heart rate and SpO_2_ levels did not change significantly before and after the procedure (Table 5).

**Table 5:** Paired t-test showing significant differences in the heart rate and no significant differences in the oxygen saturation before and after the treatment SADE: Sensory Adaptive Dental Environment; RDE: Regular Dental Environment; SpO_2_: Oxygen saturation; p<0.05: Statistically significant

Groups	Paired differences		T value	P value
	Mean	Std. deviation		
SADE heart rate before-after	-2.918	11.275	-2.227	0.029
SADE SpO_2_ before-after	0.959	1.307	6.310	0.060
RDE heart rate before-after	-0.917	10.285	-0.762	0.448
RDE SpO_2_ before-after	-0.162	0.828	-1.685	.096

Mann-Whitney U test revealed that there was no significant difference between the groups before the procedure in both behavior and anxiety levels. In the behavior assessment after the procedure, the SADE group had significantly improved behavior and significantly lesser anxiety levels (Table 6).

**Table 6:** Mann Whitney U test showing significant differences between the groups in behavior and anxiety levels SADE: Sensory Adaptive Dental Environment; RDE: Regular Dental Environment; p<0.05: Statistically significant

	Groups	Mann-Whitney	P value
Behavior before	SADE	2738	1
	RDE		
Behavior after	SADE	1972	0.001
	RDE		
Anxiety before	SADE	2138	0.148
	Control		
Anxiety after	SADE	2371	0.018
	Control		

Independent t-test revealed that there was no significant difference between the groups before the procedure in both heart rate and oxygen saturation. In the heart rate assessment after the procedure, the SADE group had a significantly lower heart rate and significantly higher SpO_2_ (Table 7).

**Table 7:** Independent t-test showing significant differences between the groups in heart rate and oxygen saturation levels SADE: Sensory Adaptive Dental Environment; p<0.05: Statistically significant

	Groups	Mean 土 SD	T value	P value
Heart rate before	SADE	92.31土11.44	-1.212	0.227
	Control	94.55土11.06		
Heart rate after	SADE	90.32土8.43	-3.606	<0.001
	Control	97.47土5.8		
SpO_2_ before	SADE	99.29土1.14	-0.985	0.326
	Control	99.47土1.02		
SpO_2_ after	SADE	99.45土2.34	4.834	<0.001
	Control	98.51土1.34		

## Discussion

Dental anxiety, or DA, is more common among young children and adolescents and encompasses physical, cognitive, and emotional aspects. It is characterized by a fear of past negative experiences during hospital visits typically affecting 5-20% of populations, and usually tends to diminish with age [16]. Previous research studies including both adults and children with developmental challenges have demonstrated the beneficial effects of multisensory environment (MSE) or SADE on mental states [17,18]. Only a few studies have been done on neurotypically healthy children to prove that MSE reduces anxiety and improves behavior.

Previously methods such as tell-show-do, modeling, verbal communication, desensitization, voice control, and hand-over-mouth exercise were used to alleviate anxiety. Advancements in multisensory approaches by employing educational principles involving additional information processing were investigated to alleviate dental anxiety in children [17]. Herein every aspect, from the softness of the dental chair upholstery to the choice of non-invasive, latex-free materials for dental instruments, is meticulously planned to ensure children have a pleasant tactile experience. Moreover, environmental temperatures are regulated for optimal comfort, understanding that extremes in temperature can be discomforting for those sensitive to certain stimuli. These elements collectively contribute to creating a more cozy and cordial tactile experience for young kids undergoing dental procedures [18].

According to Piaget's theory of cognitive development, the current study concentrated on children between the ages of four and seven; these children were in the pre-conceptual stage, which lasts from two to four years to the intuitive stage (four to seven years), during which prelogical reasoning emerges, children lack reasoning, and their thinking is self-centered and symbolic. During this period, children begin to understand things symbolically but base their reasoning more on appearances rather than logic. Positive behavior will result from an environment that calms them and externalizes the child's thoughts. In the intuitive stage, children start to form more complex images and understand concepts. However, they may encounter behavioral challenges as they navigate through this phase [19,20]. 

The Oddbodd anxiety assessment scale was used in this study to measure anxiety, and the results showed that there was significant variation in anxiety levels between the SADE and regular environments. The findings were consistent with the findings of Shapiro et al., who found that the anxious behaviors persisted longer in the SADE than in the control group [21]. In the present parallel-arm study, neurotypically healthy children were assessed. Research by Fallea et al. in Italy found that in a cross-over trial comparative study, 20% of the sample receiving treatment in RDE and 68% receiving treatment in SADE were youngsters on the autistic spectrum [22]. 

After receiving treatment in the SADE setting, children's behavior improved and their anxiety levels decreased in the current study. Similarly, in a study conducted by Cermak et al., anxiety was reduced, and behavior and cooperation increased among the children who underwent dental treatment in the SADE environment [13]. In the present study, SADE was used in the treatment room; Fux-Noy et al. conducted a study that had a SADE room as a waiting room [23]. In contrast to the present study, patients with regular waiting rooms and SADE waiting rooms did not have a significant difference in anxiety levels [23]. Using a similar concept, Kittur et al. found that when oxygen saturation levels were measured, there was a significant difference in both the groups in pre- and post-treatment levels, but no significant difference in the groups' post-intervention levels [24]. In contrast to these findings, the groups in the current investigation differed significantly after the interventions [24].

The children's heart rates were recorded using a pulse oximeter for this research. This was supported by research by Aughey et al., which demonstrated that the heart rates displayed by the pulse oximeter and electrocardiography monitor were almost exact [25]. Also in the present study, children who underwent treatment under the SADE environment significantly reduced after the treatment while in the control group, it did not. Also, there was a significant reduction in the heart rate in the SADE group compared to the control group after the treatment. Similarly, in the study done by Potter et al., heart rates were significantly less in SADE compared to the regular dental environment [24,26]. 

The present research has certain limitations. The first is that only extractions and no other invasive operations were performed or studied in the SADE setting. The second limitation was the smaller sample size and the fact that each and every assessment was carried out in a single visit. Each child that was included was categorized based on their shared behavioral patterns. Similar dental procedures with a more consistent treatment objective and a more diverse sample of dental fear and anxiety levels should be studied in future studies.

## Conclusions

The study revealed notable differences in patient experiences between the Sensory-Adaptive Dental Environment (SADE) and Regular Dental Environment (RDE). Post-operatively, participants in the SADE group exhibited better behavior and reduced anxiety compared to the RDE group.

Overall, the study suggests that sensory-friendly dental environments can significantly enhance patient experiences by helping children feel more at ease and improving their overall outcomes.
